# Genomic data integration tutorial, a plant case study

**DOI:** 10.1186/s12864-023-09833-0

**Published:** 2024-01-17

**Authors:** Emile Mardoc, Mamadou Dia Sow, Sébastien Déjean, Jérôme Salse

**Affiliations:** 1 UCA-INRAE UMR 1095 Genetics, Diversity and Ecophysiology of Cereals (GDEC), 5 Chemin de Beaulieu, 63000 Clermont-Ferrand, France; 2 Institut de Mathématiques de Toulouse, UMR 5219, Université de Toulouse, CNRS, Université Paul Sabatier, Toulouse, France

**Keywords:** Omics, Integration, System, Biology

## Abstract

**Background:**

The ongoing evolution of the Next Generation Sequencing (NGS) technologies has led to the production of genomic data on a massive scale. While tools for genomic data integration and analysis are becoming increasingly available, the conceptual and analytical complexities still represent a great challenge in many biological contexts.

**Results:**

To address this issue, we describe a six-steps tutorial for the best practices in genomic data integration, consisting of (1) designing a data matrix; (2) formulating a specific biological question toward data description, selection and prediction; (3) selecting a tool adapted to the targeted questions; (4) preprocessing of the data; (5) conducting preliminary analysis, and finally (6) executing genomic data integration.

**Conclusion:**

The tutorial has been tested and demonstrated on publicly available genomic data generated from poplar (*Populus* L.), a woody plant model. We also developed a new graphical output for the unsupervised multi-block analysis, cimDiablo_v2, available at https://forgemia.inra.fr/umr-gdec/omics-integration-on-poplar, and allowing the selection of master drivers in genomic data variation and interplay.

**Supplementary Information:**

The online version contains supplementary material available at 10.1186/s12864-023-09833-0.

## Background

In recent years, the steady development of Next Generation Sequencing (NGS) and other high-throughput technologies has led to the massive production of genomic-derived data such as genome (DNA-seq), transcriptome (mRNA-seq), methylome (BS-seq), Transposase-Accessible Chromatin (ATAC-seq), *etc.* Such data allow to investigate, with an unprecedented precision and scale, the structure and evolution of genomes and their functioning in relation to phenotypes. While different types of genome-derived data (DNA variation, gene transcription, DNA-methylation, *etc.*) provide information on specific aspects of a biological system, they are ultimately interconnected and their combination likely contains information that cannot be accessed from individual data analysis. The added-value of genomic data integration (*i.e.* the combination of the data prior to the analysis, instead of analyzing each dataset separately and then combining the results) is illustrated in the literature in reducing the complexity of multiple datasets into a single dataset, considering that a combination of datasets can contain information missing in the individual datasets [1–4]. Multi-omics data integration is increasingly being used in human [5], animals [6], and microbes [7]. Data integration is also being extensively used in plant genomic research, emerging as a promising tool in green systems biology, precision plant breeding, and other biotechnological applications [8].

Genome-derived data, and omics data in general, are heterogeneous (quantitative, such as percentages or counts, and qualitative, such as groups or classes) and produced in very large volumes, making data integration challenging. Analytical tools currently available for genomic data integration can be categorized on several levels. First, they differ in the statistical and mathematical framework, based on, e.g., dimension reduction, probabilistic models, or networks [9–12]. The integration procedures can be implemented at early, intermediate or late stages of data analysis; they can also be element or pathway-based, supervised or unsupervised, *etc.* [13, 14, 8]. Only a few are adaptable to large sets of biological features (species, individuals, tissues, genes, *etc.*) and genome-derived data such as genome, transcriptome, and methylome [15, 16]. Finally, the available tools can be categorized on the basis of the study objectives, e.g. referenced hereafter as description, selection and prediction [17–20]. In addition to the various approaches of data integration, it should also be recognized that data preprocessing and preliminary tests are essential for a successful implementation of data integration.

**Fig. 1:**
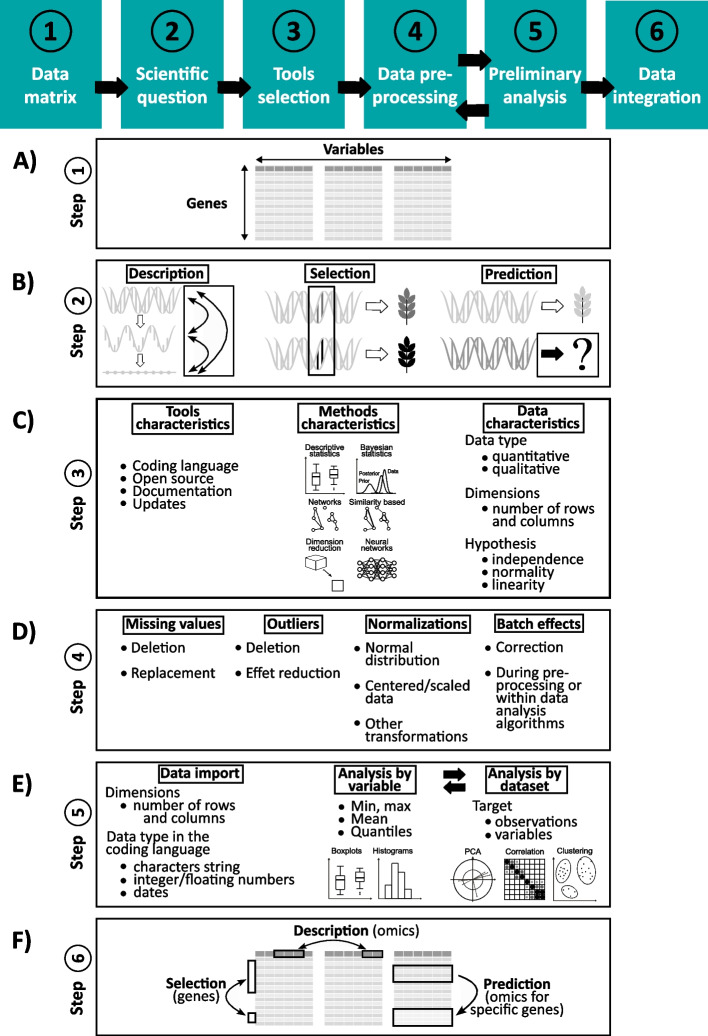
Tutorial for genomic data integration. The tutorial of best practices presents the different steps to conduct multi-omics integrations: **A**, step 1 - Constructing the genomic data matrix. **B**, step 2 - Defining a clear and precise question of interest where biological questions concern describing omics interactions and interplay, selecting biomarkers specific to a trait, or predicting phenotypes from omics; **C**, step 3 - Selecting the tool, by considering tools’ specificities such as their coding language, the accessibility or not to their source code, the quality of their documentation and frequency of their updates, their methods’ main concepts and data requirements (see Table 1); **D**, step 4 - Preprocessing data, especially to remove or impute missing values, identify then remove outliers or reduce their impact, normalize data, correct batch effects; **E**, step 5 - Pre-analyzing data, by first importing data in the expected format with the right dimensions and types, then analyzing them by variable (univariate analysis) and dataset (multivariate analysis) to reveal major insights. **F**, step 6 - Genomic data integration for data description, selection and prediction.

In combination with ever-increasing amounts of NGS-based genomic data available through public databases, data integration methods and approaches have the potential to transform our understanding of genome organization and gene regulation. To facilitate this progress, we propose a tutorial of best practices for genomic data integration, explained step-by-step and demonstrated on publicly available plant genome-derived data (from poplar [21]). The tutorial (Fig. 1) is structured in 6 consecutive steps that clarify the logical order of the procedures and allow to reach relevant conclusions, as illustrated using real datasets and exemplary research questions. It consists of (*i*) designing the adequate data matrix; (*ii*) formulating the targeted biological question; (*iii*) providing list of tools and methods for genomic data integration; (*iv*) data preprocessing, with considerations regarding missing values, outliers, normalization and batch effects; (*v*) conducting preliminary analysis where descriptive statistics and single omics analysis are necessary to properly understand the data structure and prevent misinterpretation; and finally (*vi*) performing genomic data integration with *mixOmics* on an illustrative case example.

## Results

### Genomic data matrix (Step #1)

When assessing genome-derived data from various experiments on a single individual, a group of individuals of a given species, or even on individuals from different species, one may want to gain better insight into the genomic variations and interplay of genes in the experimental design (for example, times series on a tissue exposed to a stress) used for data collection. Classically, omics data matrices consist of ‘individuals’ or ‘samples’ (biological units) arranged in lines for which available omic data (‘variables’) are listed in columns. However, genome-derived data, can be formatted as a matrix of genes considered here as the 'biological units', with genes arranged in lines and gene-related variables (e.g., diversity, expression, methylation, *etc.*) in columns (Fig. 1A). Such matrix can contain data for a single individual, multiple individuals of the same species (in additional columns), or even individuals from different species when comparing conserved genes. For the purpose of genomic data integration described here, we will consider genes as 'biological units' in lines and genome-derived data (expression, methylation, *etc.*) as 'variables' in columns. From such matrix, we propose a tutorial of the best practices dedicated to such genomic data integration following relevant steps of (*i*) the design of the data matrix, (*ii*) the identification of the biological questions, (*iii*) the choice of tools and methods for data integration, (*iv*) the data preprocessing, (*v*) the preliminary analysis with descriptive statistics and finally (*vi*) the genomics data integration.

**Fig. 2:**
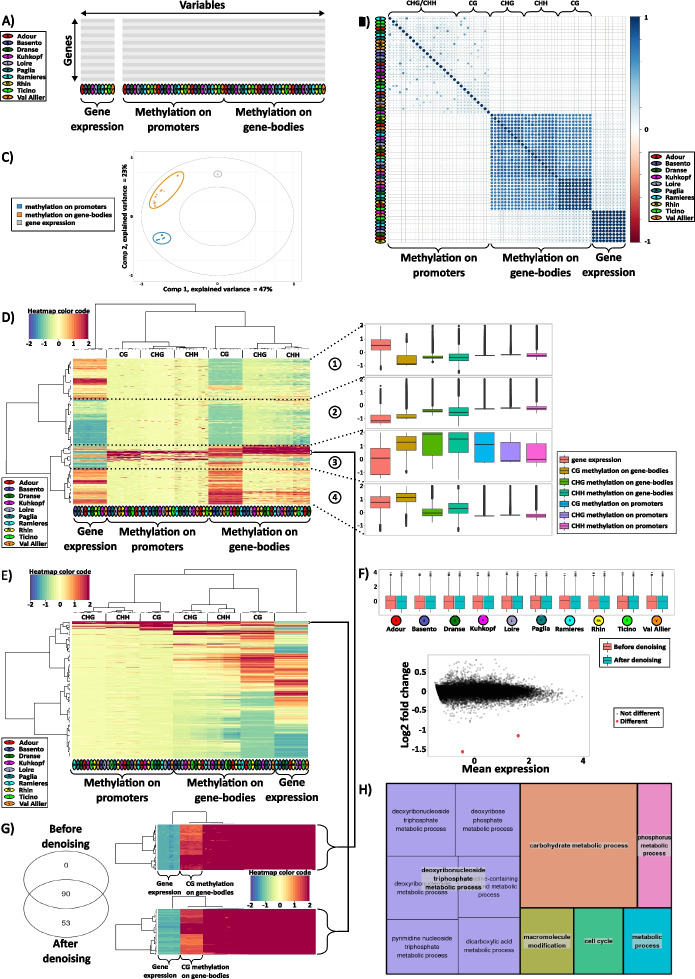
Case study of genomic (expression and methylation) data integration from 10 poplar populations. **A** Genomic data matrix with 42 950 poplar genes in lines and 70 associated variables in columns (expression and methylation for 10 populations, color code in the legend at the left). Omics variables are gene expression and DNA methylation data produced for 10 populations of poplars, as presented at the bottom left legend of the figure. Methylation data were produced for 3 contexts of methylation (CG, CHG and CHH) on two gene features (gene-body or promoter). **B** Correlation matrix of the 60 methylomics and 10 transcriptomics log-transformed variables. This figure represents Spearman’s correlation between each pair of omics variables. A high positive correlation between variables is represented by a deep blue point, a high negative correlation by a deep red point. No point means no correlation between variables. On the diagonal, correlations are by definition maximum and equals to one (*i.e.* correlated to themselves). The matrix’ variables are arranged (see color code in the legend at the right) using a hierarchical clustering with AOE (angular order of the eigenvectors) order. **C** Loading plot of omics log-transformed, centered and scaled variables on the two first components of the PCA. Omics variable are plotted on PCA’s two first principal components. For each component, the percentage of initial variance explained by this component is indicated (see color code in the legend at the left). **D** cimDiablo_v2’s result on 'non-denoised' data. Left panel: Heatmap of omics integration. Each row corresponds to one gene and each column to one omics variable. Data were centered and scaled, then a cutoff was applied in [-2,2]. According to the heatmap’s color code, blue corresponds to very low and red to very high methylated/expressed genes. Rows and columns’ dendrograms are computed by hierarchical clusterings with the Euclidean distance and Ward method to cluster together genes and omics variables sharing similar insights. Right panel: Boxplots of k cluster groups. Using the rows dendrogram, genes were divided into four groups. For each group, the average value by population for each omics variable (methylation and gene expression) is represented. **E** cimDiablo_v2’s result on 'denoised' data. Data were first centered and scaled, then 'denoised', centered and scaled a second time, before a final cutoff in [-2, 2]. **F** Comparison between 'non-denoised' and 'denoised' data for gene expression. Top panel: Boxplot of gene expression before and after the 'denoising' step. Red for 'non-denoised' data and blue for 'denoised' data. Bottom panel: MA-plot (Bland–Altman plot, where M represents the log ratio and A the mean average) of gene expression between 'denoised' and 'non-denoised' data for one of the poplar population (Adour). The x axis represents the average expression level while the y axis the log2 fold changes. Red for significant differences above |1| and black for no obvious differences. **G** Extraction of genes with extreme values (candidates) for all omics variables before and after the 'denoising' step. Left panel: Venn diagram of extracted genes before and after 'denoising'. Right panel: Heatmaps of genes with extreme values for 'non-denoised' and 'denoised' data. Gene lists are plotted using hierarchical clustering with Euclidean distance and Ward method. **H** Illustration of the Gene ontology enrichment analysis for genes with extreme values showing low expression and high methylation levels (143) after the 'denoising' step. Gene ontology enrichment has been performed using PlantGenIe (https://plantgenie.org) with *Populus trichocarpa* v3.1 as background.

In order to illustrate the use of such matrix in data integration procedures, we exploited public omics data obtained from poplar (described in [22] and [21]). The data represent transcription and cytosine methylation levels (considering the three CG, CHG and CHH contexts) for all annotated genes (considering promoter and gene-body) from ten natural poplar (*Populus nigra)* populations originating from Europe [21]. Overall, the investigated matrix consists of 70 columns (one transcriptome and six methylome columns referred to as ‘variables’ for each of the ten populations) and 42 950 lines (for annotated genes referred to as 'biological units') (Fig. 2A). This matrix will be used in the next steps of this tutorial.

### Targeted scientific questions (Step #2)

Based on published literature, genomic data are typically integrated to answer biological questions in three general categories: (1) description of the major interplay between variables (*i.e*. genomics data) or samples (*i.e.* genes), for example how DNA methylation affects gene expression at the whole genome level?; (2) selection of 'biological units' (*i.e.* genes) considered as biomarkers for a specific genomic/phenotypic response; for example groups of genes with contrasting methylation and expression patterns, or (3) variables prediction from genomic data, for example, which combination of omic variables known in one individual or species can predict the genomic behaviors of such genes in other individual or species? considering that if we have a proven association of variables in a group of individuals, then one variable can be inferred from the other in additional individuals. Overall, specifying the targeted biological question impacts the next steps of the genomic data integration (Fig. 1B).

In the use case on poplar, we demonstrate the use of the data integration tutorial by addressing all the questions above within the description-selection-prediction continuum. At the whole genome level, the objective is to unravel the general interplay between methylome and transcriptome data, *i.e.* whether DNA methylation has an impact on gene expression. At the gene level, the objective is to identify groups of genes showing contrasted profiles for both transcriptome and methylome data and then investigate their biological functions.

### Tool and method selection (Step #3)

Many tools for omics data integration have already been developed and described in published literature, and new methods keep emerging regularly. Table 1 reports 13 of the most cited tools available in R, a free software environment for statistical computing and graphics, providing lots of packages, especially in statistics and machine learning (Supplementary Table 1). Table 1 can help users to choose the most suitable tool for their particular data matrix and targeted scientific question. The selection process depends on the tools’ characteristics, capabilities, methods involved, and acceptable types of data (Fig. 1C). Among these tools, *mixOmics* can address all the scientific questions in genomic data integration referenced to as data description, selection and prediction from both quantitative and qualitative data.

**Table 1:** 13 selected R tools for omics data integration

**Tool's name**	**Scientific question**	**Method and tool's characteristics**				**Data’s characteristics**	
		**Supervised** **Unsupervised**	**Method families**	**Summary**	**Updated**	**Omics**	**Hypothesis**
**BCC **(Bayesian Consensus Clustering)	I) Description of samples' interactions	Unsupervised	Statistics	Computes a samples' clustering for each omics dataset by using a probabilistic model, then merges clusters to get a consensus cluster across omics datasets	No	Multi-omics (quantitative)	Normal distribution Different omics on the same set of samples
**iCluster **(iClusterPlus / iClusterBayes)	I) Description of samples' interactions	Unsupervised	Statistics / Dimension reduction	Starts with a latent variables regression across datasets by using a probabilistic model, then uses these joint latent variables for samples' clustering	Yes	Multi-omics (quantitative and qualitative)	Linearity assumption Normal noise distribution Different omics on the same set of samples
**JIVE** (Joint and Individual Variation Explained)	I) Description of samples/variables' interactions	Unsupervised	Dimension reduction	Decomposes each dataset in three terms: a joint effect (across datasets), an individual effect (specific to the dataset) and a noise effect	No	Multi-omics (quantitative)	Linearity assumption
**LRAcluster **(Low-Rank Approximation Cluster)	I) Description of samples' interactions	Unsupervised	Statistics / Dimension reduction	Probabilistically computes a common low-dimensional subspace across omics, then uses the K-means algorithm to cluster samples on this subspace	Yes	Multi-omics (quantitative and qualitative)	Linearity assumption Different omics on the same set of samples
**MCIA** (Multiple co-inertia analysis) (MCOA)	I) Description of samples/variables' interactions	Unsupervised	Dimension reduction	Projects each dataset on a subspace, then maximizes co-inertia between subspaces to get major information shared by datasets	Yes	Multi-omics (quantitative)	Linearity assumption Different omics on the same set of samples
**mixKernel**	I) Description of samples/variables' interactions II) Variables selection III) Phenotype prediction	Supervised Unsupervised	Dimension reduction	Transforms datasets with kernels, then applies usual dimension reduction methods	Yes	Multi-omics (quantitative and qualitative)	Datasets with the same rows or columns
**mixOmics (**with PCA, PLS, rCCA, Diablo…)	I) Description of samples/variables' interactions II) Variables selection III) Phenotype prediction	Supervised Unsupervised	Dimension reduction	Contains many matrix factorization methods for multivariate analysis and functions for data visualization. The main analysis method for one single dataset is the PCA. For two datasets or more, the main methods are the PLS and rCCA, and their extentions for discriminant analysis, variable selection ('sparse') and multi-blocks analysis	Yes	Multi-omics (quantitative and qualitative)	Linearity assumption Datasets with the same rows or columns
**moCluster **(from MOGSA)	I) Description of samples' interactions	Unsupervised	Statistics / Dimension reduction	Computes latent variables by using a PCA's extension, then clusters them and finally select the best subtype model	Yes	Multi-omics (quantitative)	Linearity assumption Different omics on the same set of samples
**MOFA **(Multi-Omics Factor Analysis)(MOFA2)	I) Description of samples' interactions III) Phenotype prediction	Unsupervised	Statistics / Dimension reduction	Factorizes datasets with a Bayesian approach to get a small number of latent factors usable for different purposes	Yes	Multi-omics (quantitative and qualitative)	Linearity assumpion
**NEMO **(NEighborhood based Multi-Omics clustering)	I) Description of samples' interactions	Unsupervised	Similarity-based	Creates one similarity matrix by dataset, then merges them and finally clusters the merged matrix by Spectral clustering	No	Multi-omics (quantitative)	Euclidean distance metric
**PINS **(Perturbation clustering for data INtegration and disease Subtyping)(PINSPlus)	I) Description of samples' interactions	Unsupervised	Similarity-based /Network	Does several clustering to identify how often samples are clustered together. Clusterings are made on different datasets, with data perturbed by adding gaussian noise, and different clustering methods are used	Yes	Multi-omics (quantitative)	Different omics on the same set of samples
**RGCCA **(Regularized Generalized Canonical Correlation Analysis)(sGCCA)	I) Description of samples/variables' interactions II) Variables selection III) Phenotype prediction	Supervised Unsupervised	Dimension reduction	Computes latent variables for each dataset by maximizing correlations within and/or between datasets	Yes	Multi-omics (quantitative and qualitative)	Linearity assumption Different omics on the same set of samples
**SNF **(Similarity Network Fusion)	I) Description of samples' interactions	Unsupervised	Similarity-based /Network	Creates a similarity matrix then an associated network for each dataset, then iteratively fuses the networks to keep only strong correlations between samples across omics	No	Multi-omics (quantitative and qualitative)	Different omics on the same set of samples Euclidean distance metric

Rows correspond to 13 selected tools and columns to the main characteristics to consider while selecting a tool for omics data integration. ‘Biological question’ describes which of the three main biological questions presented in the article the tool aims to answer. ‘Methods and tools characteristics’ details if they can be used for supervised or unsupervised analysis, at which methods’ family they belong to (statistical, dimension reduction, networks, similarity-based, artificial neural networks), summarizes its functioning, and indicates if the tool is still updated with its source code’s repository available in Supplementary Table 1. ‘Data characteristics’ describes the types of omics the tool can afford. The last column presents the main hypothesis on data, assuming they (1) follow a normal distribution (which can be tested using for instance Shapiro tests or QQ-plots), (2) share identical rows or columns (*e.g.* omics data produced on the same genes or individuals), (3) have linear interactions (*i.e.* does not consider more complex interactions such as polynomial interactions) or (4) are considered as similar according only to the Euclidean distance (*i.e.* does not consider other metrics of similarities such as correlations)

In the use case on poplar, we chose *mixOmics*, an open-source tool developed in the R programming language for omic data integration purposes. *mixOmics* contains several functionalities based on dimension reduction methods to explore one dataset or integrate two or more datasets, depending on the biological questions and data types available. The dimension reduction methods aim to extract the main sources of variation from datasets that are usually very large (*i.e.,* have potentially thousands of rows and/or columns). This tool is associated with an active forum (https://mixomics-users.discourse.group) and a documented website (http://mixomics.org/) that are available to help throughout the whole analysis, from the choice of the integrative and graphical functions to the interpretation of outputs. Moreover, *mixOmics* contains many functions to also analyze the data, which are generally derived from Principal Component Analysis (PCA) and Projection to Latent Structures (PLS) regression methods. These methods aim to factorize initial datasets/matrices (into: Components x Loadings + Residuals) to reduce datasets’ dimensions while retaining the components with the main information from the initial omic variables. *mixOmics* also allows to display results with many graphical functions. “Sample plots” display observations (*i.e.* referred to as individuals or samples in the current study), while “variable plots” display omics variables (*i.e.* genomic data in the current study).

### Data preprocessing (Step #4)

Genomic data matrix must be preprocessed to take into account, when necessary, missing values, outliers, normalization and batch effects (Fig. 1D). Missing values can be handled by deletion (deleting each row or each column containing missing values or defining a threshold proportion of missing data over which columns-rows are deleted) or replacement (by 0, by the minimum of the values, by the average of other values, the median or a quantile and ultimately, by imputation), as summarized in [23]. Outliers, defined as an unusual value compared to the rest of the dataset (either due to error, or due to unique behavior of an investigated individual for a specific variable), can be (1) deleted from the dataset if considered as errors, (2) separated but not excluded if they represent an interesting behavior of the biological system (one analysis can focus on the outlier values, and another one on the rest of the data), or (3) transformed (normalized) in order to keep the outliers in the data matrix but reduce their effect in the analysis. If the user decides to reduce the outliers’ effects, one solution is to use the logarithmic normalization of the variable. This transformation has a minor impact on low values but strongly reduces high values, while retaining the rank (relative order) of data points. However, log-transformation is not applicable to zero values, in which case errors are returned. To overcome this problem, a constant (and relatively small) value is usually added to all zeros, e.g. (+1) as we did in the case study. The appropriate increment applied to zeros prior to the log-transformation needs to be carefully considered with the biological meaning/interpretation of such transformation. In the case study, we choose +1 for log2 transformation of the transcriptomic data, because we consider that genes with TPM (Transcripts Per Million) ≤ 1 are not expressed and replacing zeros with +1 would therefore not affect the results. However, data transformations also change the scale of the variables, making it more difficult to interpret the new values. Generally, data normalization methods consist of transforming raw data, e.g. in order to obtain values that follow a Gaussian distribution to be used with parametric tests, or to center and/or scale heterogenous variables facilitating their comparison [24]. Data normality (*i.e.* a match with the Gaussian distribution also called normal distribution) can be tested for instance with Shapiro tests or QQ-plots [25]. Normalizations, as all other data transformations, should always be considered with regards to the effect on the interpretability of the results (*e.g.* does a normalization procedure that changes positive values to negative ones affect the subsequent computational steps or result interpretation?). Finally, batch effects are effects caused by a non-biological factor during any step of data production (including the preparation of biological material), undesirably biasing sub-groups of data [26]. They are generally identified during preliminary data analysis and can be corrected by functions such as *ComBat* (from R package *sva*, https://rdrr.io/bioc/sva/man/ComBat.html) and *removeBatchEffect* (from R package *limma*, https://rdrr.io/bioc/limma/man/removeBatchEffect.html).

In the illustrative case study, the data needed preprocessing due to the presence of missing values (i), and outliers (ii). In order to reduce the missingness (i), only genes with at least one expression and one methylation value were considered. Hence, from the 42 950 genes annotated in the poplar genome (v3.1), we kept 31 040 genes (72%). Secondly, we fixed the missing value cut-off to 10%, allowing only 10% of missing values for the whole dataset, further trimming the gene set to 28 267 (67% of the annotated genes). In addition, we created another dataset where no missing value was allowed for the investigated variables (genomics data), consisting of 24 962 genes (58% of the annotated genes). The outlier issue (ii) was addressed with a logarithm function, which reduced the impact of extreme values (Supplementary Fig. 1). In the case of the methylation variable (Supplementary Fig. 1A), promoter methylation remained to be strongly impacted by a few genes with extreme values, even after the log-transformation. Methylation on gene-bodies is more variable, with few values approaching zero (*i.e.* no methylation). Gene-body methylation is higher in the CG-context compared to the other sequence contexts, with a large group of highly methylated genes. Regarding transcription (Supplementary Fig. 1B), genes are divided in two groups, non-expressed *vs.* highly expressed genes.

### Preliminary data analysis (Step #5)

This step consists of clarifying variable types, data dimension, and associations between rows ('biological units', *i.e.* genes) or columns (‘variables’, *i.e.* genomic data) of each dataset (Fig. 1E). On a single dataset level, useful methods for such preliminary analysis are (1) the Principal Component Analysis (PCA) allowing to extract the major information signal contained in the dataset, (2) the correlation matrix to highlight strongly associated pairs of variables, and (3) clustering methods such as the hierarchical clustering or the K-means algorithms to identify the most similar pairs of individuals or variables.

In our case example, the preliminary data step consisted of displaying the matrix of correlations (Fig. 2B) between each pair of variables (columns). Most variables were strongly correlated among the ten individuals (representing different populations) of poplar, indicating little variation between populations, especially for transcription and gene-body methylation in the CG context. Much weaker correlation was observed between individuals for promoter methylation; nonetheless, a cluster analysis consistently grouped all individuals in blocks of different variables. Weak correlations were observed between some omics blocks (*i.e.* between expression and gene-body methylation, or between promoter methylation and gene-body methylation). Another way to rank the genomic effects was highlighted with the PCA (Fig. 2C). Typically, a 'score plot' is used to display samples (‘biological units’, here genes) on the first couple of principal components, in order to identify clusters of samples and possible outliers (standard PCA plots). Here, we used a 'loading plot' to map variables on these components, in order to identify how omics variables are clustered. On the first principal component, data are divided in two groups, methylation *vs.* expression. On the second component, data are divided in two other groups, promoter methylation *vs.* gene-body methylation and expression. Overall, such preliminary data analysis revealed that the contribution of the omic variables to the variation in the dataset follows this order, from the highest to the lowest effects, (1) the type of omics (expression *vs.* methylation), (2) the methylated compartment of genes (promoters *vs.* gene-bodies) and (3) the methylation contexts (CG *vs.* CHG and CHH), and finally (4) the different populations (individuals). Moreover, it revealed that gene-body methylation is more variable than promoter methylation, especially in the CG context. No general association between gene methylation and expression has been observed with this analysis at the whole genome level.

### Genomic data integration (Step #6)

In addition to pairwise genomic data comparison [22], multiscale genomic data integration aims at characterizing hidden and potentially complex interactions between different omics data in order to provide a better comprehensive understanding of cellular and biological processes (Fig. 1F). The PLS regression function in *mixOmics*, allowing to compute a linear combination of omics variables to extract a smaller number of ‘components’ retaining data variability, is generally recommended for two omics datasets analyzed (see description in Step #3). Since our methylation data is more complex and was described into six variables (two gene partitions - promoters and gene-bodies - and three sequence contexts - CG, CHG, CHH), we opted for ‘*block.pls’*, a generalization of the PLS for more than two datasets (called ‘blocks’). This choice was directly made based on the insights gained from the previous preliminary analysis, particularly the different strength of association between gene expression and the various methylation subtypes. We used the *block.pls* function in its ‘regression’ mode, with methylation as explicative data and expression as explicated data, to focus on the impact of DNA methylation on gene expression. First, a ‘design matrix’ needs to be set up, considering the data being integrated. The ‘design matrix’ contains weights between all pairs of blocks with values multiplying each covariance between two blocks: a higher value for interactions between pairs of blocks (*i.e.* values multiplying covariance between two blocks), where a high value is assigned for interactions of high interest, and a low value is assigned for interactions of low interest [27]. In the use case on polar, the chosen weights are 0 for each block with itself, 1 for each methylome block with the transcriptomics block and 0.1 for each pair of methylome blocks. These weighting values were specifically chosen to focus on the interactions between methylome and transcriptome data, but also to take into account the interactions between the different methylome blocks/contexts or gene compartments. Such interactions are well-represented on a clustered heatmap, similar to the output of the graphical function *cimDiablo* from *mixOmics* (for multi-blocks PLS regressions). However, this function is currently applicable only for the discriminant methods *block.plsda* and *block.splsda*, and not for the non-discriminant methods *block.pls* and *block.spls*.

To overcome this limitation, here we developed in the current study *cimDiablo_v2,* a new function based on *cimDiablo* from mixOmics for non-discriminant *block.pls* and *block.spls* objects, which could take into account a ‘denoising’ step, and is publicly available at https://forgemia.inra.fr/umr-gdec/omics-integration-on-poplar. The ‘denoising’ step uses the components and loadings’ matrices from *block.(s)pls*. The components are computed to keep the essential information from the initial data, mainly the variability conserved across omics variables. Therefore, the variability that is specific to one row (*e.g.* sample, individual, gene *etc.*) or one column (omics variable), referred to as ‘noise’, is extracted from the matrix of components and placed into a matrix of residuals, *cimDiablo_v2*. The ‘denoising’ step displays the data without the residuals (*i.e.* noise), corresponding to the matrix product of the components and loadings matrices, referred to as ‘denoised’ matrix. Most of *cimDiablo_v2* parameters are the same as for *cimDiablo*, with only a few specific to *cimDiablo_v2*. These correspond to the binary parameters to apply for data transformations: ‘denoise’ (to ‘denoise’ data as described above), ‘scale2’ (to center and scale data after the ‘denoising’ step) and ‘cutoff ’ (to set all values higher than 2 to 2 and all values lower than -2 to -2).

We ran *cimDiablo_v2* on the poplar data with and without the ‘denoising’ step (Fig. 2D, Fig. 2E, Supplementary Fig. 2). The graphical function of *cimDiablo_v2* (like *cimDiablo*) allows to display a heatmap where hierarchical clustering reveals the interactions between rows (*i.e.* genes) and between columns (*i.e.* variables) and the heatmap of rows-columns interactions. Without the ‘denoising’ step, the overall picture resulted in clustering of samples by omics variables (promoter methylation, gene-body methylation and gene expression), Fig. 2D. Gene-bodies appeared to be clearly more methylated than promoter regions especially in the CG context, confirming our previous results in the preliminary analysis (step #5). More marked methylation differences between populations were observed for non-CG contexts, especially for the CHH context which drives more methylation differences between populations. We then split the dendrogram into 4 groups (or clusters) of genes (rows) defining different typologies of genes in their methylation-expression regulation (Fig. 2D), delivering the following gene categories, (1) high expression level and low methylation levels for promoters and gene-body in the three methylation contexts (2) low expression level, low CG gene-body methylation and moderate non-CG gene-body and promoter methylation levels (3) high methylation levels and moderate to low expression levels, and finally (4) high expression and CG gene-body methylation with moderate methylation in other features and contexts.

In order to focus on the general trends or ‘master’ genomics regulators-drivers (*i.e. *genes that are regulated though expression-methylation, or omics in general, interplay) in all populations, we used the ‘denoising’ step aiming at removing the variability that is specific to one row, variable or population (Fig. 2E). After the ‘denoising’ process, there is less variation between genomic variables per gene. At the whole genome level, this ‘smoothed’ effect of the ‘denoising’ step on the clustering heat map (Fig. 2E) can help identifying general trends or master regulators-drivers, i.e. genes with expression-methylation interplay shared between any investigated individuals, development stage, etc. At the gene level, it also allows to identify only genes with extreme omic profiles. To assess the impact of the ‘denoising’ step, we compare the ‘non-denoised’ and ‘denoised’ data (Supplementary Fig. 3). Interestingly, the two ‘denoised’ and ‘non-denoised’ datasets look quite similar, especially for expression data suggesting that only a few values have been changed. To precisely quantify those changes, we display MA plots to assess differences between the ‘denoised’ and ‘non-denoised’ data. Only a few genes show expression differences after the denoising step (Fig. 2F). However, for methylation data, more differences are observed after the ‘denoising’ step as more marked variability was initially reported between the populations, especially for promoter and non-CG gene-body methylation (Supplementary Fig. 4). Overall, we recommend the use of the ‘denoising’ step to assess both genome-wide and gene level interplays between genomics data in order to identify general trends as well as master regulators-drivers, or in a broader sense, to remove the variance between biological replicates in experimental setups.

In order to identify genes where the omic pattern is indicative of an association between DNA methylation and gene expression, we used the ‘denoised’ matrix containing no missing value (24 962 genes). Interestingly, only few genes (143) showed a contrasting pattern between expression and all methylation variables simultaneously, *i.e.* highly methylated and lowly expressed genes in all studied populations (Fig. 2G). Comparison between ‘non-denoised’ and ‘denoised’ datasets revealed that all highly methylated and lowly expressed genes identified before ‘denoising’ (90 genes) are found after the ‘denoising’ procedure, suggesting that ‘denoising’ does not lead to signal loss. However, 53 additional genes were specifically identified only in the ‘denoised’ data, suggesting that the procedure improves sensitivity of signal detection. This outcome aligns with expectations for a procedure that removes signals appearing only once (in a single omic variable or population). Gene Ontology (GO) analysis on the identified set of highly methylated and lowly expressed genes revealed enrichment in functions related to involvement in carbohydrate, cell cycle, phosphorus and metabolic processes (Fig. 2H). Among these genes, we identified *Di19* (Drought induced 19) that enhance drought tolerance in transgenic poplar plants [28]. First identified in *Arabidopsis, Di19* has been characterized as a new type of transcription factor, directly up-regulating the expression of *PR1, PR2* and *PR5* in response to drought stress [29]. The results of our integrated omic analysis may suggest that the expression of *Di19* in poplar trees could be associated with DNA methylation, suggesting a possible epigenetic regulation of this gene that can be explored in future studies to be potentially exploited in breeding schemes especially in response to drought stress.

## Discussion

The constant development in sequencing methods and strategies, as well as reduction in cost, allows access in the public domain to genomic data from many plant species. How to make proper use of these data to unravel plant genome organization and regulation in different environmental contexts remains a key question for both fundamental and applied research, especially in characterizing genomic makers of crop adaptation to constraints to be exploited in breeding schemes. A tutorial of best practices when conducting genomic data integration has been proposed from a specific data matrix consisting on genes (rows) and genomic variables (columns) in order to unravel the genomic interplay of genes of several individuals of given species or individual from distinct species. The proposed tutorial applied here on the integration of genomic data can also be applied on any omics data taking into account non genome-derived data (*i.e.* proteome, metabolome, phenotype…) with individuals (*i.e.* accession) instead of genes, in lines. The tutorial has been illustrated on a case example from methylation and expression data obtained from 10 poplar populations from Europe to reveal genomic interplay (between expression and methylation) at the whole genome and gene levels. The proposed tutorial is divided in 6 steps: data matrix design, biological question, tools selection, data preprocessing, preliminary analysis and finally genomic data integration.

Regarding the targeted biological question, genomic data integration is generally conducted for the (1) description of the major interplay between variables, (2) selection of genes considered as biomarkers, (3) prediction of some variables from genomic data. Regarding the first type of questions, one may be interested in global interplay between the omics data, for example correlations between genomic variables. When addressing the second type of questions, specific groups of genes showing similar or contrasted behaviors on one or several variables are selected. For example, users can look for genes from which genomic variation control a specific phenotype (resistance to a disease or temperature stress). Finally, regarding the third type of questions, phenotype prediction from omics data consists in transferring knowledge of omics-phenotypes interactions across individuals (plant varieties, animal species, *etc.*). Hence, it has been used in medicine to predict diseases evolution from cohorts [30], or in agronomy to predict yield (grain) production in cereals [31].

Regarding the tools available to conduct omics data integration, scientific articles already offered reviews or benchmarks of omics data integrative tools and methods to help choosing the best integrative approach. Here, we report from eight review articles [11, 13, 18–20, 32–34], 13 most cited tools in R programming (Table 1), although popular tools such as t-SNE [35] or UMAP [36], usable for non-linear dimension reduction, were not cited by these articles. The 13 tools rely on distinct methods to consider with caution depending on the biological question addressed, (1) descriptive and inferential statistics, (2) dimension reduction, (3) network and/or (4) similarity-based approaches. The descriptive statistical approaches [37] use mathematical means such as the mean, median, variance, standard deviation and graphics such as boxplots to describe the data. Statistic tests are part of the inferential statistics [38] aiming to validate or not a hypothesis on data’s probabilistic distribution. Bayesian approaches [39] also belong to the inferential statistics and assume, before data analysis, that these data follow a chosen probabilistic distribution called the *prior*, then compute the *posterior* distribution by fitting the *prior* to the data. Regarding dimension reduction methods [12, 40], they aim to extract the largest part of the information contained in the data and store it in new data with lower number of dimensions. Once the omics integration tools (13 proposed) and associated methods (6 described) have been selected based on the methodological principles previously described, data need to be treated prior integration.

Regarding the data preprocessing prior to integration, four major steps have been identified, concerning missing values, outliers, normalization-transformation and the batch effects. To help to conduct data preprocessing, some algorithms have been developed for testing and comparing different methods, for instance with the R packages *missMethods* (https://rdrr.io/cran/missMethods/), *outliers* (https://rdrr.io/cran/outliers/), *bestNormalize* (https://rdrr.io/cran/bestNormalize/) and *bapred* (https://rdrr.io/cran/bapred/), overall allowing to manage the four preprocessing steps.

The preliminary analysis consists of conducting a descriptive investigation of the data in order to avoid misinterpretation of the results based on basic graphics to clarify variables’ types, data dimension, basic associations between rows (samples) or columns (variables) of each dataset, *etc.* Knowing variables’ ranges and distributions is very useful for data preprocessing. Indeed, users must decide whether values should be centered and/or scaled according to data ranges and distributions. Common methods are to look for minimum, median, mean and maximum values, or to derive graphs such as boxplots or histograms, when possible. Outliers are in general explicitly detected and visible by boxplots representation or in histograms when there is enough data. Before data integration, preliminary analysis of each dataset separately is strongly recommended in order to avoid mis- or over-interpretation of the results.

Other workflows from the scientific literature are usually divided in three steps: omics data, data preprocessing, simple and integrative analysis [17, 34, 41]. Compromises have been done in this study to offer a tutorial of best practices with enough information to integrate genomic datasets. Omics integrative methods for more specific purposes are presented in reviews and benchmark articles [10, 12, 16, 17, 42–47]. Moreover, some steps of the data preprocessing (redundancy, heterogeneity, *etc.*) are not presented here, but available for example in [33]. Many benchmark articles [18, 19, 32, 34] also discuss concrete effects of omics integration tools on the same datasets. From our knowledge, only [27] presents a workflow starting from the biological question of interest, that we consider should be the starting point of any workflow for conducting omic data integration. This workflow also has the advantage to be cyclic, as multi-omics integration naturally lead to new questions and then additional analysis. In complement to the previous articles, we provide here a step-by-step procedure allowing to conduct genomic data integration that we made publicly available at https://forgemia.inra.fr/umr-gdec/omics-integration-on-poplar.

Methylome and transcriptomics data from poplar have been integrated following the proposed tutorial to assess the impact of DNA methylation on gene expression, and to identify candidate genes with contrasted profiles across methylome and transcriptomics data. We developed a new function ‘*cimDiablo_v2’* allowing the possibility to ‘denoise’ data to maximize the identification of ‘real’ or ‘strong’ omics interplay where managing noise in omics data is still a challenge [48, 49], especially to remove exclusively variability with no biological meaning. Our proposed method does not focus on removing biological meaningless information, but more precisely on removing isolated variability, *i.e.* gene variability specific to one omics variable, while keeping gene variability shared across omic variables or individuals. The case example has permitted to obtain several results with (1) the highest effect that structure the investigated omics data being the data type (transcriptomics *vs.* methylomics), then the gene feature (gene-bodies *vs.* promoters), the methylation context (CG *vs.* CHG *vs.* CHH methylation), and finally the population (10 populations from western Europe); (2) there is more variability on methylation on gene-bodies than promoters, especially the CG methylation; (3) there is no general trend between gene methylation and expression at the whole genome level; and (4) genes with contrasted expression-methylation profiles across omics variables are involved in carbohydrate, cell cycle, phosphorus and metabolic process and key functions involved in response to stresses, an important trait for the adaptation of perennial species (poplar) to different geographical environments. Overall, the use case illustrates the power of genomic data integration to identify genes driving key traits through specific genomic (expression-methylation) interplay that can be precisely identified, prior their exploitation in crop management and breeding schemes.

## Conclusion

We propose a step-by-step tutorial for genomic (*i.e.* DNA-based) data integration illustrated on a case example on poplar plant consisting in (1) designing a data matrix, (2) defining a specific biological question, (3) selecting the appropriate tools, (4) performing data preprocessing, (5) conducting preliminary analysis, and (6) performing multi-omics integration. In addition, we developed *cimDiablo_v2,* a new function based on *cimDiablo* from *mixOmics* for non-discriminant *block.pls* and *block.spls* objects available at https://forgemia.inra.fr/umr-gdec/omics-integration-on-poplar and exploitable on any type of omics data.

## Materials and method

### Genomic data analyzed

*Genome* - Genomic data analyzed here have been retrieved from [21] and [22] for DNA methylation and gene expression respectively. The samples used here are initially from [21] where authors analyzed a collection of 241 genotypes of *P. nigra* populations using RNA-seq in order to assess gene expression. Recently, [22] retrieved a subset of 10 populations (*i.e.* 20 genotypes) from [21] on the same tree individuals and the same sampling time. This subset of 10 populations were analyzed using WGBS (methylome), together with the transcriptome data from [21] in order to assess the role of epigenetic regulation in driving tree species evolution and adaptation. *Methylome* - DNA methylation analysis was done in [22] on the same sample powders from [21]. Briefly, for DNA methylation, genomic DNA was extracted using a cetyl trimethyl-ammonium bromide (CTAB) protocol and whole-genome bisulfite sequencing was performed in accordance with the procedure described by [50]. Reads from sequencing were then mapped against the poplar v3.1 reference genome and methylation call realized with *BSMAP *[51] using default options, delivering 3 datasets for the 3 methylation contexts. The *Methylkit* (v1.18.0) and *genomation* (v1.32.0) R packages were used for the annotation of DNA methylation data in genomic regions (promoters and gene body). Hence, the methylome dataset consists in three methylation contexts on two genes’ regions, overall producing six methylation variables by population. Methylome dataset is expressed as the number of methylated cytosines x number of methylated cytosines / number of cytosines, called rbd (read by density), [22]. *Transcriptome* - For transcriptomic (gene expression) dataset, from [21], RNA-seq was carried out with Illumina Hiseq2000 platform. Reads were mapped on the *Populus trichocarpa* v3.0 reference genome using *bowtie2* (v2.4.1) [52]. Raw counts were then normalized by Trimmed Mean of M-values (TMM) from edgeR (v3.26.4) [53] as described in [21].

### Genomic data integration

*Genomic data matrix* - The design matrix consists of 42 950 polar genes in lines and 70 associated variables in columns. *Defining a specific biological question *- At the whole genome level, the objective is to unravel the general interplay between methylome and transcriptome data, *i.e.* if DNA methylation has an impact on gene expression. At the genes level, the objective is to identify groups of genes showing contrasted profiles for both transcriptome and methylome data and then investigate their biological functions. *Selecting appropriate tool* - We use *mixOmics* for genomic data integration associated with an active forum (https://mixomics-users.discourse.group) and a documented website (http://mixomics.org/). *Performing data preprocessing* - To deal with missing values, only genes with at least one expression and one methylation values and less than 10% of missing values for the whole dataset were considered. To reduce outliers’ impact, data were log-transformed with log_2_(1+x), where x represents methylation / expression values and 1 the constant number added when dealing with zero values. *Conducting preliminary analysis* - A matrix of correlations was performed on preprocessed data without missing values using Spearman correlation and AOE (Angular Order of the Eigenvectors) criteria for variables clustering. PCA analysis was conducted on preprocessed data with missing values, firstly centered and scaled, to compute 2 components. *Performing multi-omics integration* - *mixOmics block.pls* regression was conducted on 6 methylation on 1 expression blocks, with a design matrix composed of 1 between expression and methylation, 0.1 between methylation blocks and 0 within each block, and finally 2 components computed by block. Data were first centered and scaled in block.pls, then several cimDiablo_v2 results were obtained depending on if data are ‘denoised’, centered and scaled a second time and/or cut in [-2, 2]. For cimDiablo_v2 plots, a hierarchical clustering was used with the Euclidean distance and Ward method. Master regulators-drivers were selected both on ‘non-denoised’ and ‘denoised’ data, by selecting genes with all methylation values higher than 1 and all expression values lower than -1. For the comparison of ‘non-denoised’ and ‘denoised’ data, log2 fold changes cut-off above |1| were applied using MA-plot, and a Bland-Altman plot for visual representation of genomic data.

### Supplementary Information

**Additional file 1:** **Supplementary Figure 1.** Histograms of methylomics and transcriptomics’ logged distributions from one poplar (Adour) population. **Supplementary Figure 2.** Omics data integration with cimDiablo_v2. **Supplementary Figure 3.** Boxplots of k cluster groups in each poplar population for gene expression and methylation. **Supplementary Figure 4.** Comparison between 'non-denoised' and 'denoised' data for methylation in gene-body and promoter for CG, CHG and CHH contexts.

**Additional file 2:** **Supplementary Table 1.** 39 R tools for multi-omics data integration. **Supplementary Table 2.** Gene annotation of 'no-denoised' and 'denoised' candidate genes. Annotation information have been retrieved from PlantGenIE (https://plantgenie.org/) with Populus trichocarpa v3.1 as a reference. The column ‘common_before_and_after_denoising’ indicates whether the gene is shared between 'denoised' and 'no-denoised' data or not (TRUE/FALSE).

## Data Availability

Genomic data integration code in R is made publicly available at https://forgemia.inra.fr/umr-gdec/omics-integration-on-poplar. Datasets produced in the current article are made available at https://entrepot.recherche.data.gouv.fr/privateurl.xhtml?token=d946bb29-4698-4bee-9c6b-c2d98558ca8a. Plants material is directly involved in the study with omics data derived from natural poplar populations described in the material and method section of the current manuscript.
