# Supplementary material for: Genomic data integration tutorial, a plant case study
Source: BMC Genomics. 2024 Jan 17;25:66. doi: 10.1186/s12864-023-09833-0 (PMC10792847; doi:10.1186/s12864-023-09833-0)

**Supplementary Figure 1: Histograms of methylomics and transcriptomics’ logged distributions from one poplar (Adour) population.**

Histograms (y-axis for gene counts) for **A-** methylation (x-axis) normalized in rbd and logged for gene body (left) and promoter (right) of genes by contexts (CG, CHG or CHH), and **B**- gene expression (x-axis) normalized in TMM and logged.

**A)**


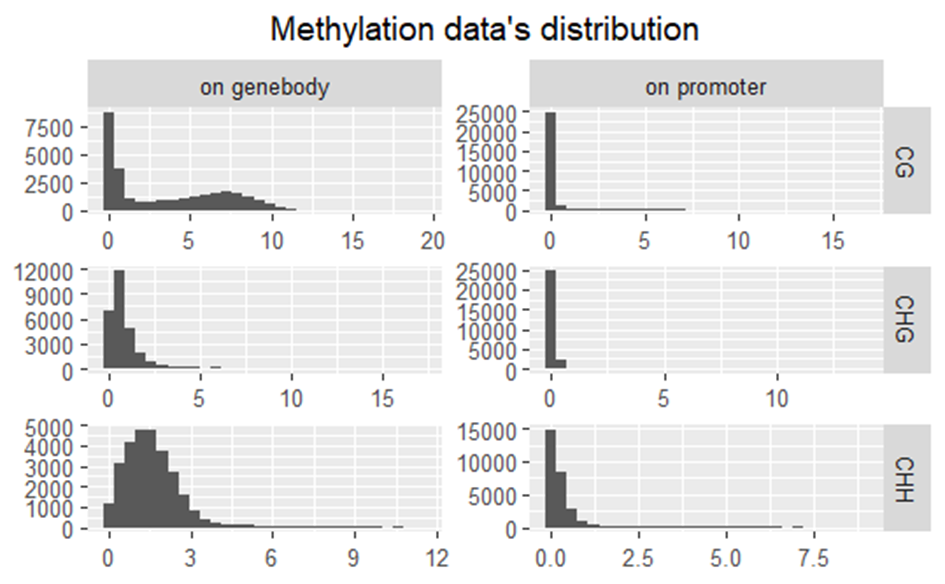


**B)**


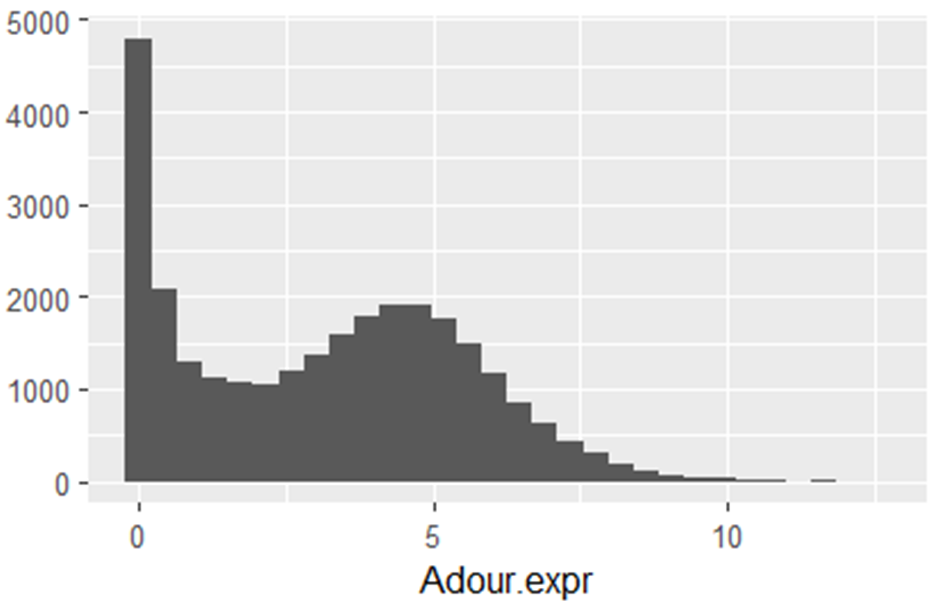


**Supplementary Figure 2: Omics data integration with cimDiablo_v2.**

Each row corresponds to one gene and each column to one omic variable. Omics variables are gene expression and DNA methylation data produced for 10 populations of poplar, as presented in the down left legend of each sub-figure. Methylomics data were produced for 3 contexts of methylation (CG, CHH, CHG) on two gene features (gene-body or promoter). Heatmaps’ values are computed following steps consisting of (1) center and scale a first time of initial data (done by default with block.(s)pls), (2) denoise data as presented in the current manuscript (Mardoc et al. 2023), (3) center and scale a second time the data, (4) apply a cutoff in [-2, 2]. According to the heatmap’s color code, blue corresponds to very low and red to very high methylated or expressed genes. Rows and columns’ dendrograms are computed by hierarchical clusterings with the euclidean distance and Ward method to cluster together genes and omics variables sharing similar profiles. **A-** represents centered and scaled data, without any denoising nor second centering/scaling steps, and data cut in [-2, 2]. **B-** represents data centered and scaled before being denoised then cut in [-2, 2]. **C-** represents data centered and scaled before being denoised, then centered and scaled a second time, and finally cut in [-2, 2]. **D-** represents data centered and scaled before being denoised, then centered and scaled a second time, but without any cutoff.


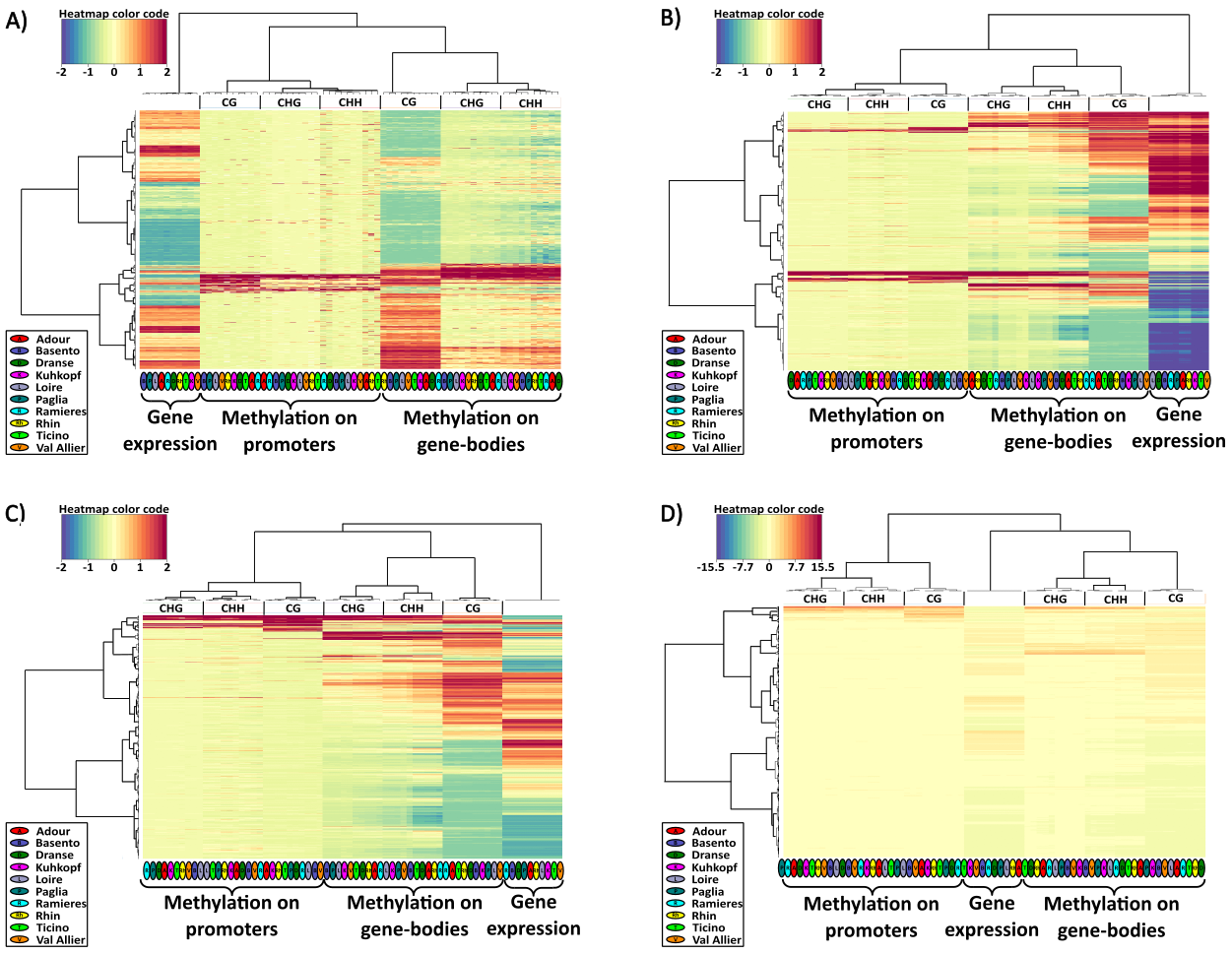


**Supplementary Figure 3: Boxplots of k cluster groups in each poplar population for gene expression and methylation**.

**A-** Boxplots of omics variations with non-denoised data. According to the row dendrogram, genes were divided into four clusters. For each cluster gene expression and methylation levels (for gene-body and promoter in the three methylation contexts) are represented as boxplots for the 10 studied populations. **B-** Boxplots of omics variations with denoised data. According to the row dendrogram, the optimal number of k clusters was set to seven. The level of gene expression or DNA methylation is shown for each cluster in each population.

**A)**

**
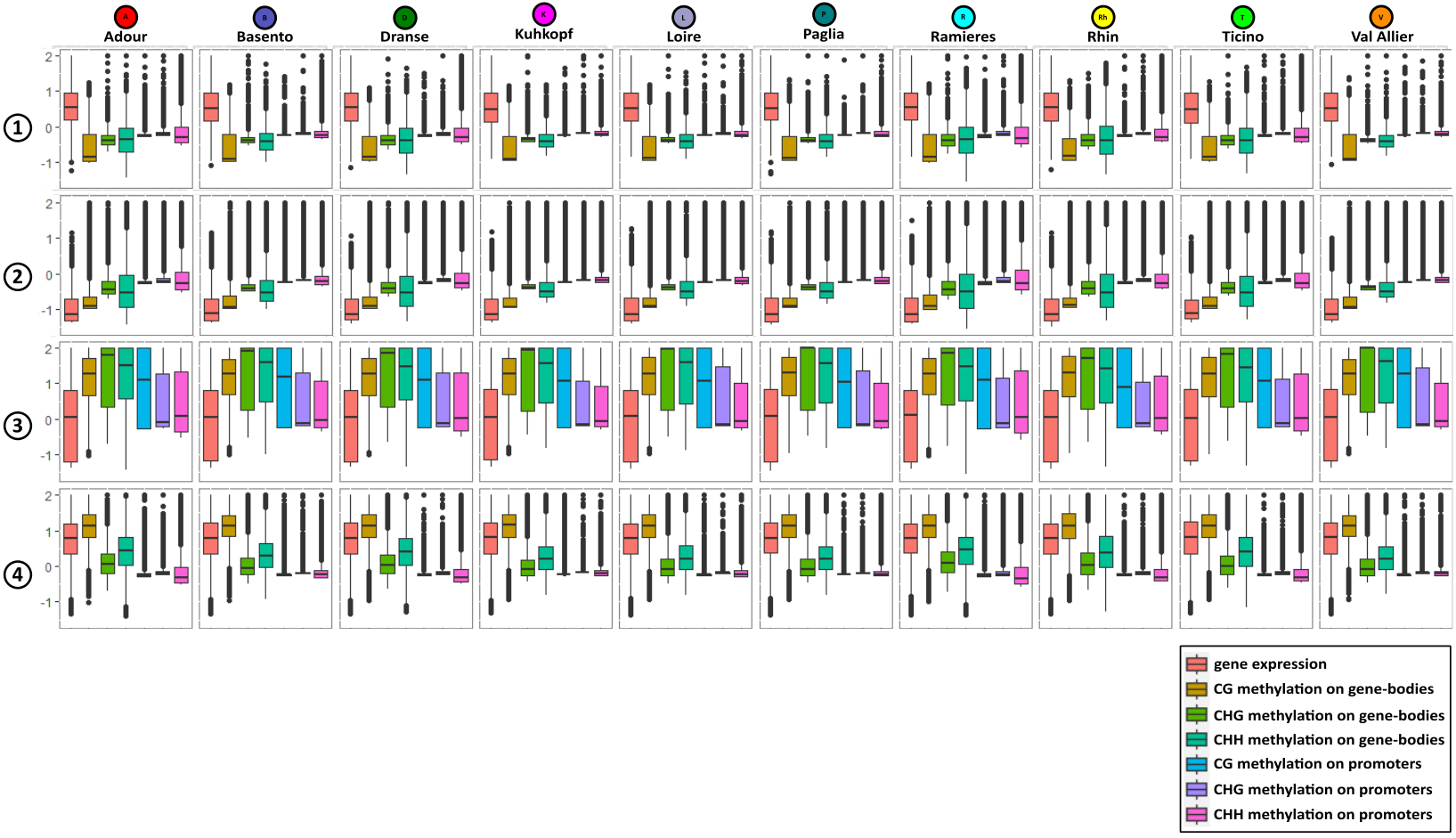
**

**B)**


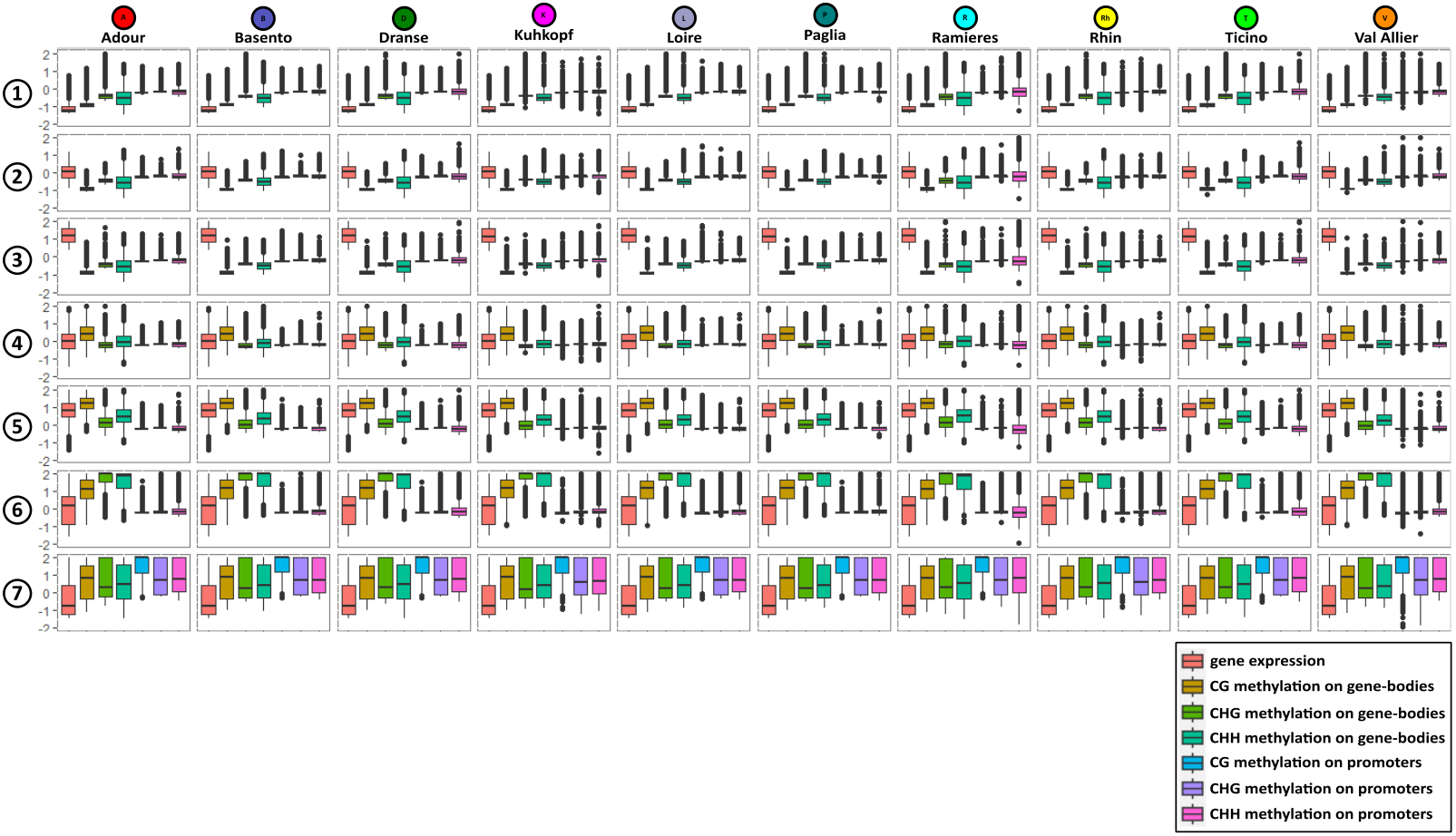


**Supplementary Figure 4:** **Comparison between non-denoised and denoised data for methylation in gene-body and promoter for CG, CHG and CHH contexts**.

**A-** Boxplots of methylation levels between non-denoised (red) and denoised (blue) data in the ten studied populations (gb for Gene-body methylation; prom for promoter methylation). **B-** MA-plot of one polar (Adour) population for DNA methylation between non-denoised and denoised data. The x axis represents the average expression level while the y axis the log2 fold changes. Red for significant differences above |1| and blacks for no obvious differences.

**A)**


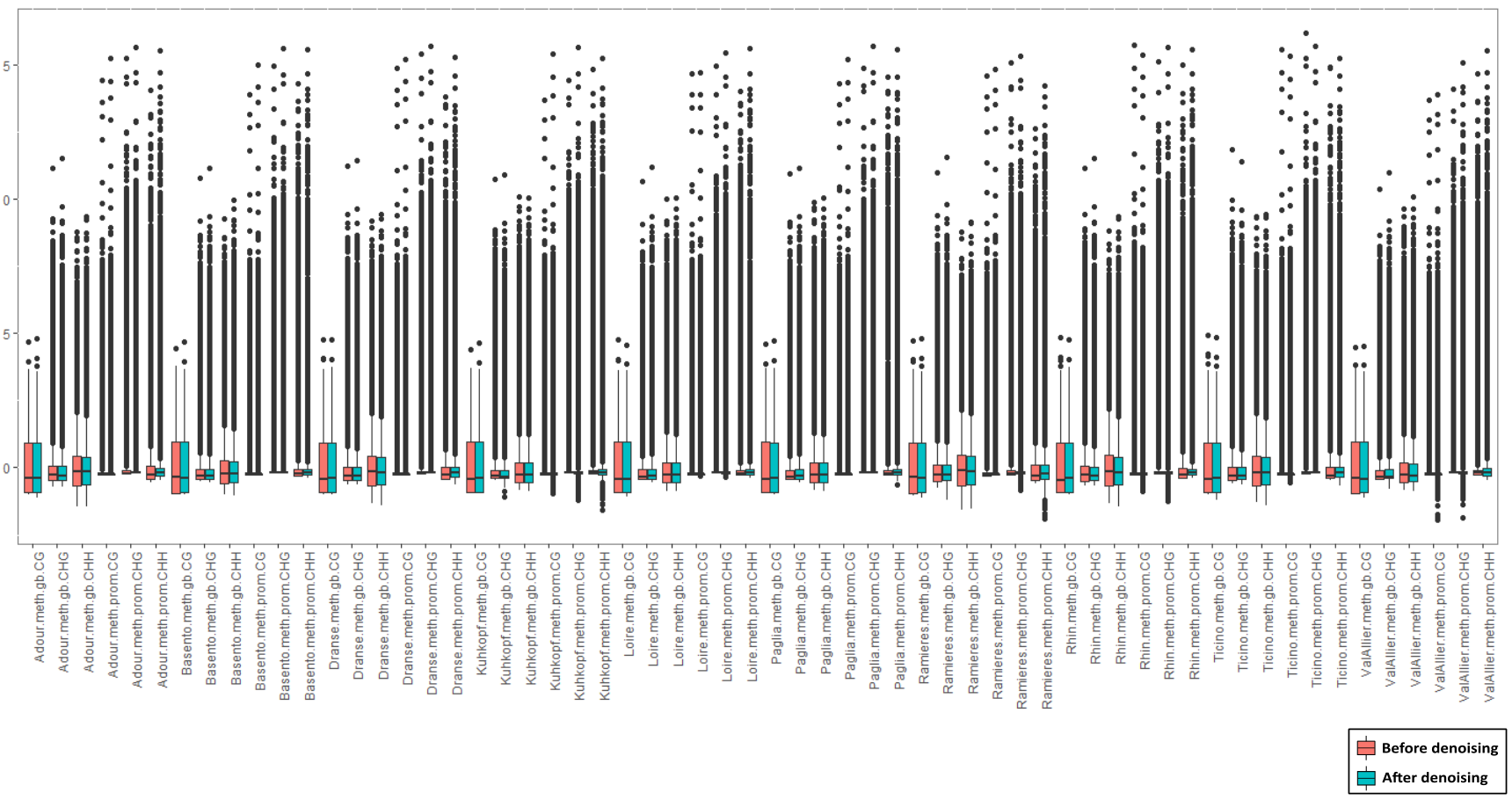


**B)**


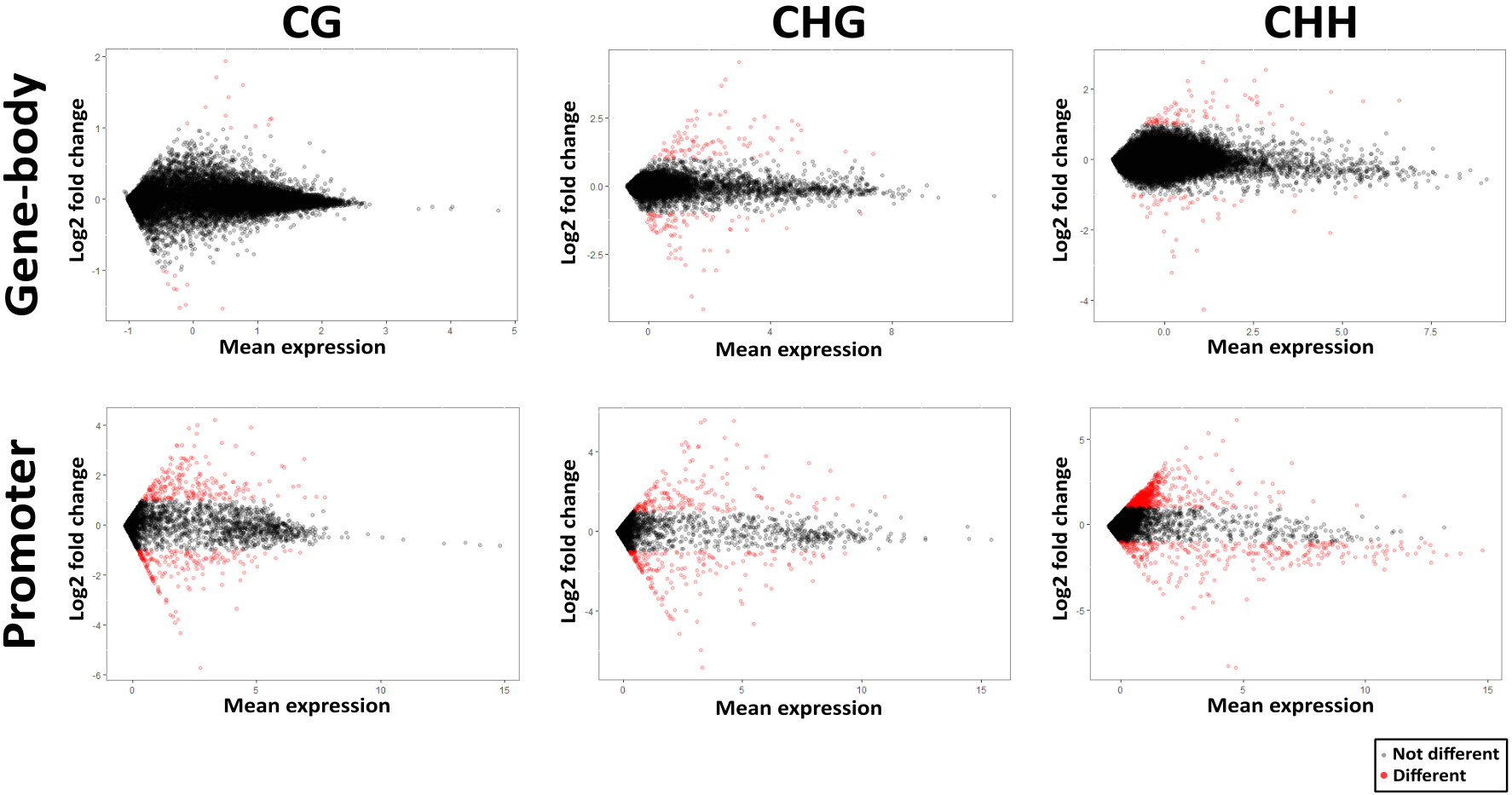

Supplement: Supplementary file 1 — Additional file 1: Supplementary Figure 1. Histograms of methylomics and transcriptomics’ logged distributions from one poplar (Adour) population. Supplementary Figure 2. Omics data integration with cimDiablo_v2. Supplementary Figure 3. Boxplots of k cluster groups in each poplar population for gene expression and methylation. Supplementary Figure 4. Comparison between 'non-denoised' and 'denoised' data for methylation in gene-body and promoter for CG, CHG and CHH contexts. [file 12864_2023_9833_MOESM1_ESM.docx]
